# COXPRESdb in 2015: coexpression database for animal species by DNA-microarray and RNAseq-based expression data with multiple quality assessment systems

**DOI:** 10.1093/nar/gku1163

**Published:** 2014-11-11

**Authors:** Yasunobu Okamura, Yuichi Aoki, Takeshi Obayashi, Shu Tadaka, Satoshi Ito, Takafumi Narise, Kengo Kinoshita

**Affiliations:** 1 Graduate School of Information Sciences, Tohoku University, 6-3-09, Aramaki-Aza-Aoba, Aoba-ku, Sendai 980–8679, Japan; 2 Institute of Development, Aging, and Cancer, Tohoku University, Sendai 980–8575, Japan; 3 Tohoku Medical Megabank Organization, Tohoku University, Sendai 980–8573, Japan

## Abstract

The COXPRESdb (http://coxpresdb.jp) provides gene coexpression relationships for animal species. Here, we report the updates of the database, mainly focusing on the following two points. For the first point, we added RNAseq-based gene coexpression data for three species (human, mouse and fly), and largely increased the number of microarray experiments to nine species. The increase of the number of expression data with multiple platforms could enhance the reliability of coexpression data. For the second point, we refined the data assessment procedures, for each coexpressed gene list and for the total performance of a platform. The assessment of coexpressed gene list now uses more reasonable *P*-values derived from platform-specific null distribution. These developments greatly reduced pseudo-predictions for directly associated genes, thus expanding the reliability of coexpression data to design new experiments and to discuss experimental results.

## INTRODUCTION

Large-scale gene coexpression is a powerful tool to elucidate gene functional modules. Over the past 10 years, gene coexpression has been extensively used, especially in plant science, for the prioritization of genes with a particular function of interest (1–5). In addition to microarray technologies, recent RNAseq data produced by high-throughput sequencing technologies are available for the construction of coexpression systems (6–8). The accumulation of RNAseq data opens the possibility to apply the coexpression approach even to non-model species. In animal science, coexpression information is largely used as part of the supporting information to predict protein–protein interactions, such as with the STRING (9), IMP (10) and Funcoup (11) databases. A limited number of databases are now focusing on coexpression information as the main content, such as GeneFriends (12), GeneMANIA (13) and STARNET2 (14). Gene coexpression has a long range to search, even for weak functional associations. Thousands of genes are meaningfully coexpressed for one cellular function (15). However, this long-range characteristic becomes problematic when a researcher wants to search for only directly associated genes, as in protein–protein interaction relationships. To focus on searching for directly associated genes with functions that can be successively examined by experiments, such as for protein binding or for gene disruption/overexpression, there are two fundamental refinement points for gene coexpression. The first point is the construction of more reliable, high-quality coexpression data, and the second point is the accurate estimation of the quality of the coexpression data. In addition, an appropriate data representation framework is valuable to distinguish true gene associations from false ones. Even if the average predictive performance is low, appropriate data representation with accurate reliability scores for each prediction will safely guide users to select the appropriate genes to design new experiments.

We have been continuously developing the coexpression database, COXPRESdb (coexpression database), providing high-quality coexpression data with new metrics (16,17) and various analysis frameworks (18–20). For example, when a user is interested in a particular gene, the coexpressed gene list for the target gene is available from the coexpressed gene page. When a user is interested in a particular function, the EdgeAnnotation tool, NetworkDrawer tool and the GO network page easily bring all the genes associated with the function along the coexpressed genes. The information and the tools can be used for the 11 species (human, mouse, rat, monkey, dog, chicken, zebrafish, fly, nematode, budding yeast and fission yeast) in COXPRESdb, and users can evaluate the reliability of the coexpression information by inspecting coexpression in orthologous genes. Some other features and the overview of COXPRESdb are available at http://coxpresdb.jp/overview.shtml.

In this paper, we report our recent improvements of COXPRESdb, in terms of two refinement points. For the first point, we newly prepared RNAseq-based gene coexpression data for three species (human, mouse and fly), and increased the number of microarray experiments to nine species. Increased number of coexpressed gene pairs supported by multiple platforms indicates steady improvement of the coexpression data set. For the second point, we refined the data assessment procedures for each coexpressed gene list, which now uses more accurate *P*-values based on platform-specific null distribution. These developments minimize pseudo-predictions for directly associated genes, thus expanding the utility of coexpression data for designing new experiments and discussing experimental results.

## OVERVIEW OF THE LATEST COEXPRESSION DATA

### New coexpression data

In addition to the microarray-based coexpression, we newly prepared RNAseq-based coexpression data, which have high potential, especially for genes with low expression levels. The construction of the RNAseq-based coexpression data has been slightly modified from our previous method applied to Arabidopsis (8). We first selected the RNAseq data entries using the SRAdb R/Bioconductor package (21,22) with the following options: platform = ILLUMINA, library_strategy = RNA-Seq or TRANSCRIPTOMIC. The selected RNAseq entries were downloaded from the DDBJ Sequence Read Archive (23). Then, the FASTQ data were mapped onto the NCBI RefSeq mRNA sequences (24), using Bowtie2 (25). To cover the genes with lower expression level, runs including large number of reads were selected (total mapped counts >10 000 000), resulting 5626, 3746 and 754 runs for human, mouse and fly, respectively. The mapped counts were summed for each gene model and used as the gene expression value. Genes with lower levels of expression; i.e. with average counts across all runs <30, were omitted. After conversion to a base-2 logarithm with a pseudo-count of 1, quantile normalization was applied to the data from each experiment, and the average expression levels were subtracted from each gene for each experiment. Using all experiments at once, Pearson's correlation coefficients for each gene pair were calculated, and these values were transferred to the Mutual Rank (MR) value (16), which is the geometric average of asymmetric ranks in coexpressed gene lists.

With the updated microarray-based coexpression, using the recent public microarray data stored in ArrayExpress (26), Table 1 shows the summary of the platforms for coexpression data in COXPRESdb. Note that we did not update the three platforms (Hsa, Mmu, Rno) that have more than 20 000 samples. These three platforms are relatively old and already include sufficient numbers of samples, and thus we regarded these coexpression data as having reached the quality plateau. The procedure for the calculation of the microarray-based coexpression is the same as in our previous report (16).

**Table 1.** Coexpression data provided in COXPRESdb

Species	Dataset ID	Platform	Number of genes	Number of samples (v5.0)	Number of samples (v6.0)
*Caenorhabditis elegans*	Cel	A-AFFY-60	17 256	1034 (c2.0)	1528 (c3.0)
*Canis lupus*	Cfa	A-AFFY-149	16 211	377 (c1.0)	636 (c2.0)
*Drosophila melanogaster*	Dme	A-AFFY-35	12 626	3336 (c2.0)	4741 (c3.0)
*Drosophila melanogaster*	Dme2	RNAseq	13 099		754 (c1.0)
*Danio rerio*	Dre	A-AFFY-38	10 112	1126 (c2.0)	1727 (c3.0)
*Gallus gallus*	Gga	A-AFFY-301	13 757	1024 (c2.0)	1301 (c3.0)
*Homo sapiens*	Hsa	A-AFFY-44	19 803	73 083 (c4.0)	73 083 (c4.0)
*Homo sapiens*	Hsa2	A-AFFY-141	19 788	6865 (c1.0)	12 640 (c2.0)
*Homo sapiens*	Hsa3	RNAseq	19 816		5636 (c1.0)
*Macaca mulatta*	Mcc	A-AFFY-145	15 781	675 (c1.0)	875 (c2.0)
*Mus musculus*	Mmu	A-AFFY-45	20 403	31 479 (c3.0)	31 479 (c3.0)
*Mus musculus*	Mmu2	RNAseq	19 453		3746 (c1.0)
*Rattus norvegicus*	Rno	A-AFFY-43	13 751	27 481 (c3.0)	27 481 (c3.0)
*Saccharomyces cerevisiae*	Sce	A-AFFY-47	4461	2693 (c1.0)	3819 (c2.0)
*Schizosaccharomyces pombe*	Spo	A-AFFY-47	4881	111 (c1.0)	224 (c2.0)

### Increased number of common coexpression edges

For drawing the coexpression network, we used the top three coexpressions for every gene as the coexpression edges. This coexpression threshold, three, was selected to achieve simpler virtualization of the network. Some coexpression edges repeatedly appear in different platforms, indicating the strong, highly reliable association of the gene pair. Such edges are highlighted in orange in NetworkDrawer, which is the coexpression network-drawing tool in COXPRESdb. An increase in the number of such commonly observed coexpression edges could be an indicator of favorable growth of the database. Therefore, we first summarized the number of edges commonly observed in the different platforms (Supplementary Data). To compare the platforms for different species, we used orthologous genes provided from HomoloGene (27). As expected, the number of commonly observed edges increased along with the higher data version. Especially, multiple platforms for the same species presented higher rate of common coexpression edges. For example, among the 7784 coexpression edges in the Dme platform, 1112 edges also appear in the Dme2 platform, but only 70 edges appear in the Hsa platform. When we focus on the platforms for different species, the number of commonly observed edges seems to reflect the species phylogeny. This phenomenon is the result of the number of homologous genes, as well as the diversity of the transcriptome. The ratio of the edges commonly observed in the other platform reached 28% in the human Hsa2 platform, whereas just 6% of the total edges in the Cel platform (nematode) and Gga platform (chicken) are reproduced in the other platforms. Although the absolute ratios of common edges vary among the platforms, the ratios of each platform have steadily increased through this improved version of the database.

## EVALUATION OF THE QUALITY OF COEXPRESSED GENE LIST

### Refinement of the statistical evaluation of each coexpressed gene list

The existence of commonly observed coexpression edges (Supplementary Data) is a naive method to evaluate the non-randomness of each coexpressed gene pair. However, this method causes several problems in the statistical evaluation of the coexpression quality as follows; (i) Since the network edges in COXPRESdb are restricted to the top three coexpressed gene relationships from every guide gene for simplicity, they may reflect only a part of the gene coexpression relationships. (ii) Each platform includes different number of genes, and thus statistical significant of top *k* gene can be different (*k* = 3 was employed as the network edge). (iii) The all possible reference platforms should be compared to select the best one.

To overcome these problems, we partially updated the quality measure. Instead to compare the top three coexpression edges, we compared two coexpressed gene lists, namely, the coexpressed gene list from a guide gene *g* of interest (*list_g_*) and that from a reference guide gene *r* (*list_r_*), which is the orthologous gene (or the same gene in the same species) in other platforms. To quantify the concordance of the two lists in a weighted manner, we introduced the measure *COXSIM* (8,20):

(1) \begin{equation*} {\rm COXSIM}_{k}^{g}(r)= \sum \nolimits _{i=1}^{k} n(i, list_{g}, list_{r})/\sum \nolimits _{i=1}^{k} i, \end{equation*}

where $n(i,list_g ,list_r )$ is the number of genes in the top *i* genes in *list_g_* having corresponding genes (orthologous genes in the case of different species comparisons, and the same gene in the case of different platform comparisons in the same species) in the top *i* genes in *list_r_*. To take into account different number of genes for each platform, we have modified *k* in the F1 from 100 to the top 1% of all genes in *list_g_*. To select the best reference guide gene, we checked all possible reference guide genes. The reference guide gene set *R* is composed of all available orthologous genes for different species. When multiple platforms are available for the species including the guide gene *g*, the same gene in the other platforms is also included in the reference guide gene set *R*. The *COXSIM* values are calculated between the target guide gene *g* and every reference gene *r* in *R*. The reference gene that gives the maximum *COXSIM* value is regarded as the best reference guide gene:

(2) \begin{equation*} {{\it maxCOXSIM}_{1\% }^g} = \max _{r \in R} \left[ {{\rm COXSIM_{1\% }^g} (r)} \right] \end{equation*}

To assess statistical significance, the ${{\it maxCOXSIM}_{1\% }^g}$ value is compared with the null distribution generated under the same number of genes in *list_g_*. On the COXPRESdb, the significance level, which we call the *supportability*, is shown as the number of stars according to the following *P*-value thresholds: 1E-04 for one star, 1E-16 for two stars and 1E-32 for three stars (example: http://coxpresdb.jp/cgi-bin/coex_list.cgi?gene=7535&sp=Hsa). Detail explanation for *supportability* is shown in the COXPRESdb help page [http://coxpresdb.jp/help/supportability/].

**Figure 1.**
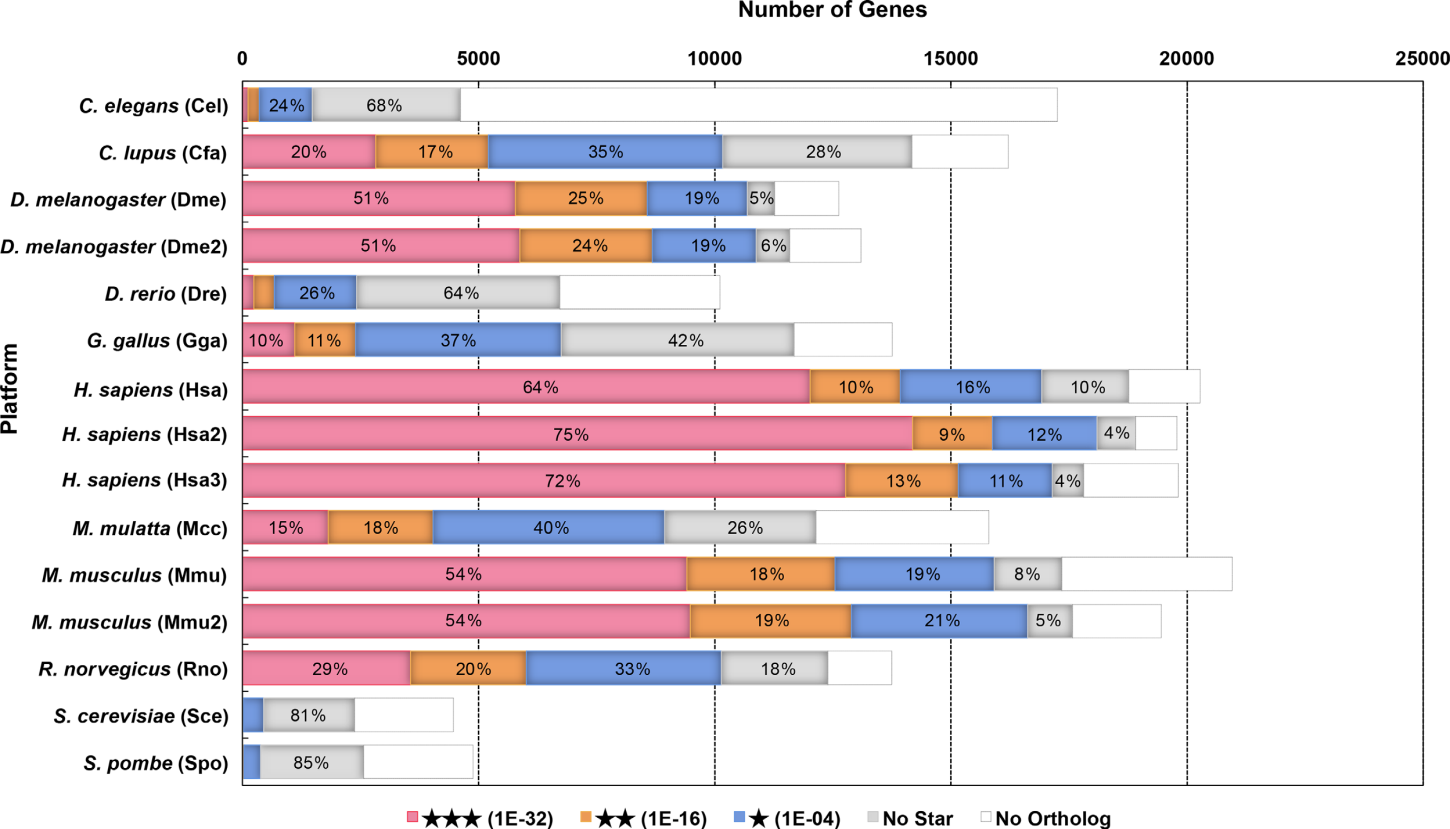
Number of guide genes for each supportability level. Supportability levels are represented as stars, where no star is the lowest and a triple star is the highest. Numbers in the bars indicate the percentage of each supportability level in each platform. Genes without any reference genes in the other platforms are shown as a blank box.

The numbers of genes at each significance level are summarized in Figure 1. Human, mouse and fly show higher populations of high supportability level genes than the other species, reflecting the fact that these species have multiple platforms that are most suitable to prove the reliability of the coexpressed gene lists. Note that ‘No star’ does not always mean unreliable. ‘No star’ is also available for coexpression specifically observed in the species, or it may just result from the lack of an appropriate reference platform. Even so, for general purposes, a high supportability level of coexpression is recommended to prioritize genes. As the human platforms have strong coexpression support (Figure 1), the human coexpression data in COXPRESdb are one of the most important resources for human research.

## OTHER DEVELOPMENTS

The *supportability* is not applicable to nematode and yeasts, because they do not have close high-quality reference platform. To give an opportunity to investigate platform validity, we introduced three independent scores, (i) Reproducibility score, (ii) Codon score and (iii) GO score. Reproducibility score is based on the supportability and reference adequateness. Codon score represents general consistency between coexpression and genome signature. GO score shows consistency between coexpression and GO annotation (see the detail of these scores). All assessment scores (Reproducibility score, Codon score and GO score) for every coexpression data set, including the previous versions, are shown in the Download page in COXPRESdb [http://coxpresdb.jp/download.shtml]. These scores for the previous coexpression versions were calculated based on the latest versions of coexpression, RefSeq sequences and GO annotations, respectively. Although these three measures are not conclusive to evaluate coexpression data, these measures gives an opportunity to consider the use of nematode and yeasts data. In addition, all the three measures support continuous refinement of coexpression data in COXPRESdb.

In addition to the individual representations of the high-quality coexpression data in COXPRESdb, bulk download functions (http://coxpresdb.jp/download.shtml), RDF by SPARQL (http://coxpresdb.jp/sparql) and API returning the JSON format (see http://coxpresdb.jp/help/API.shtml), are available to easily combine with other large-scale data.

## SUPPLEMENTARY DATA

Supplementary Data are available at NAR Online.
